# Supplementary material for: Force transmission through the inner kinetochore is enhanced by centromeric DNA sequences
Source: eLife. 2025 May 29;14:RP105150. doi: 10.7554/eLife.105150 (PMC12121997; doi:10.7554/eLife.105150)
Supplement: Figure 1—source data 1. [file elife-105150-fig1-data1.zip › Figure 1-source data 1.pdf]

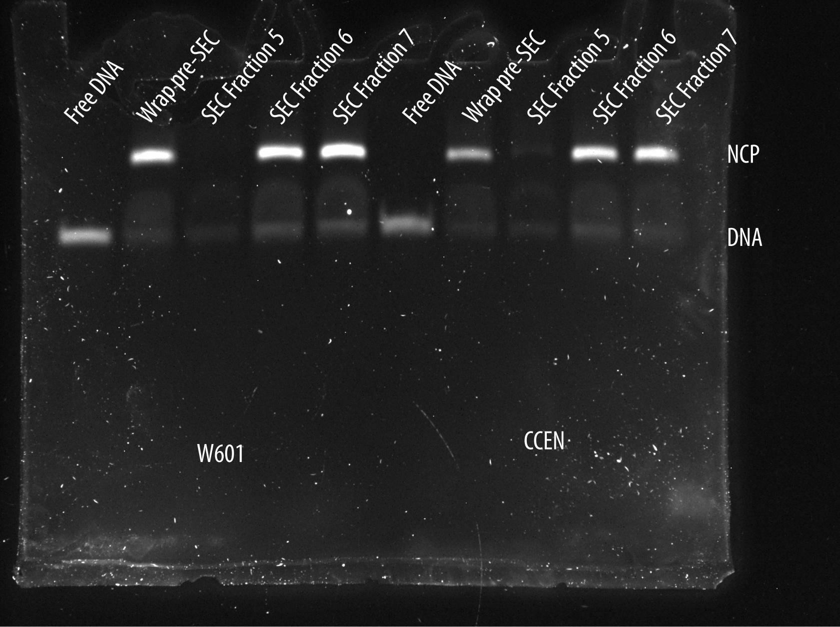
Free DNA
Wrap pre-SEC
SEC Fraction 5
SEC Fraction 6
SEC Fraction 7
Free DNA
Wrap pre-SEC
SEC Fraction 5
SEC Fraction 6
SEC Fraction 7
NCP
DNA
W601
CCEN
