# Supplementary figures and images for: Force transmission through the inner kinetochore is enhanced by centromeric DNA sequences
Source: eLife. 2025 May 29;14:RP105150. doi: 10.7554/eLife.105150 (PMC12121997; doi:10.7554/eLife.105150)

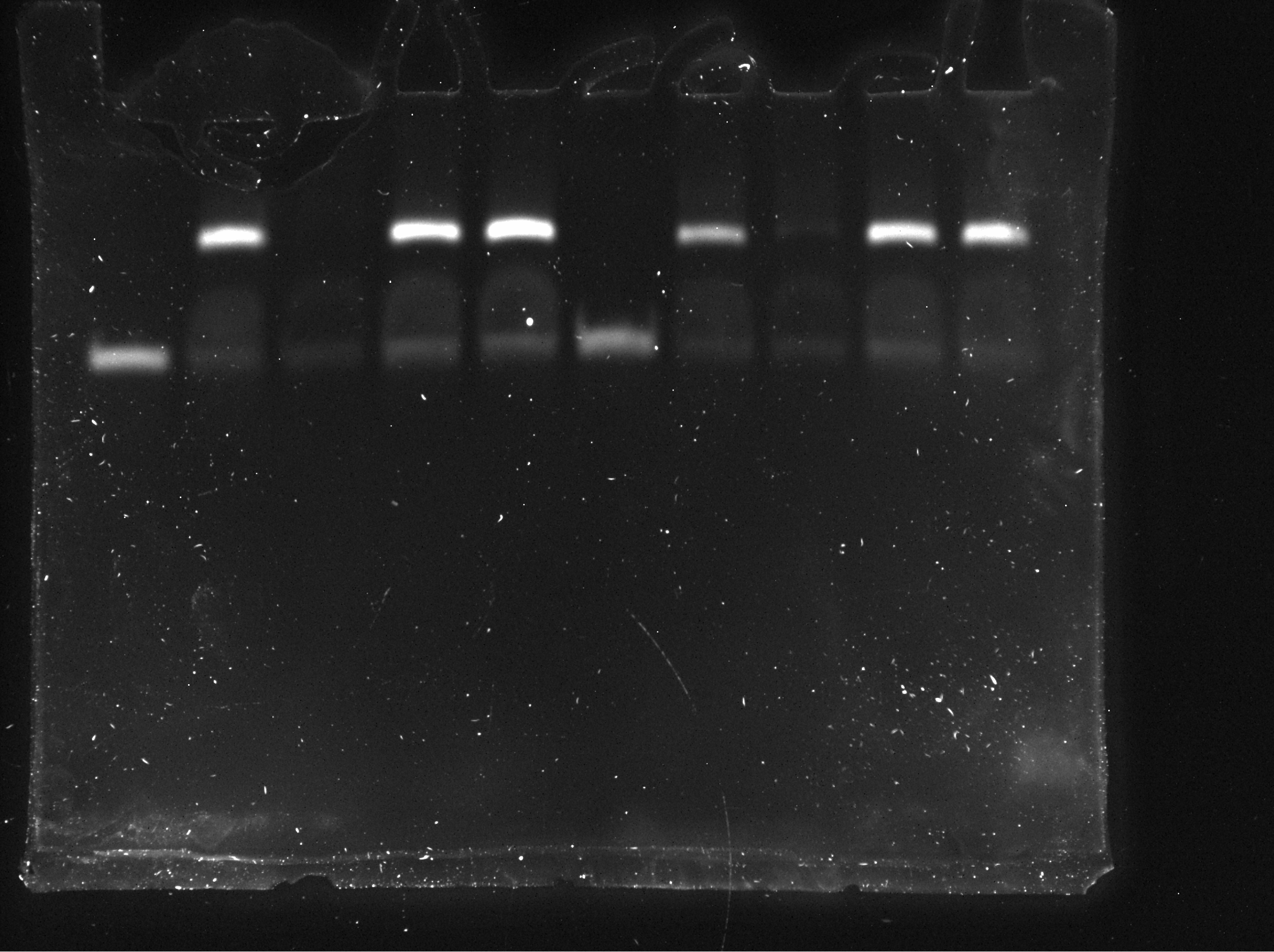

Supplement: Figure 1—source data 2. [file elife-105150-fig1-data2.zip › Figure 2-source data 2.tif]
